# Neutrophil-Lymphocyte Ratio and Urine Albumin-Creatinine Ratio As Indicators of Microvascular Complications in Type 2 Diabetes Mellitus: A Cross-Sectional Study

**DOI:** 10.7759/cureus.75196

**Published:** 2024-12-06

**Authors:** Sai K Upadhyayula, Sharath Ubaru, P. Raajeshwi, C.N. Ajavindu, Anirudh B Rao

**Affiliations:** 1 Internal Medicine, Kempegowda Institute of Medical Sciences, Bengaluru, IND; 2 General Practice, Kempegowda Institute of Medical Sciences, Bengaluru, IND

**Keywords:** diabetic eye disease, diabetic microangiopathy, diabetic peripheral neuropathy (dpn), endocrinology and diabetes, internal medicine (general medicine)

## Abstract

Background

Type 2 diabetes mellitus (T2DM) is associated with a high risk of developing microvascular complications such as diabetic nephropathy, diabetic neuropathy (DN), and diabetic retinopathy (DR), leading to significant morbidity. Early detection of these complications is crucial for improving patient outcomes. Neutrophil-lymphocyte ratio (NLR) and urine albumin-creatinine ratio (UACR) show promise as cost-effective and accessible biomarkers for the early detection of microvascular complications in T2DM. Their integration into routine care could enhance risk stratification, facilitate timely interventions, and improve patient outcomes, reducing the burden of diabetes-related morbidity. However, their clinical utility in diabetic populations remains underexplored.

Objective

The study aims to evaluate the predictive value of NLR and UACR for microvascular complications, specifically DN and DR, in patients with T2DM.

Methods

This cross-sectional study included 130 patients diagnosed with T2DM undergoing routine investigations at the Department of General Medicine, Kempegowda Institute of Medical Sciences, Bengaluru. NLR and UACR, along with other secondary variables were measured, and their associations with DN and DR were analysed using various statistical tests to assess the viability of these biomarkers in predicting microvascular complications in clinical practice.

Results

UACR emerged as a strong predictor for both DR and DN. UACR achieved an accuracy of 91% for DR (area under the curve (AUC) 0.97) and 81.5% for DN (AUC 0.90). NLR showed 85% accuracy for DR (AUC 0.87) and 75% accuracy for DN (AUC 0.851). However, NLR was not a significant predictor in multivariate analyses, suggesting that other variables may affect its predictive ability. Logistic regression analyses identified UACR, duration of diabetes, and glycosylated haemoglobin (HbA1C) as significant predictors of microvascular complications. The models had adjusted R² values of 0.751 for DN and 0.881 for DR.

Conclusion

The study highlights the predictive value of NLR and UACR in detecting microvascular complications, particularly DN and DR, in patients with T2DM. UACR demonstrated superior utility compared to NLR, underscoring its clinical relevance in early screening for complications. Additionally, glycaemic control and diabetes duration were significant predictors, emphasising the importance of comprehensive monitoring in preventing diabetic complications. Further research is warranted to explore the role of NLR in larger, more diverse populations.

## Introduction

Type 2 diabetes mellitus (T2DM) has reached epidemic levels globally, with the World Health Organisation (WHO) noting an increase in prevalence among adults over 18 from 4.7% in 1980 to 8.5% in 2014 [1]. According to the 2019 Diabetes Atlas by the International Diabetes Federation, approximately 463 million people currently live with diabetes, a number projected to rise to 700 million by 2045, particularly in developing countries [2]. This escalating prevalence poses a significant burden on healthcare systems due to diabetes-related complications. In India, there were nearly 102.26 million cases of diabetes in 2016, with a prevalence of 7.8% (7.9% in males and 7.5% in females) [3].

T2DM is characterised by chronic hyperglycaemia, resulting from irregularities in insulin production and function, which disrupts the metabolism of carbohydrates, fats, and proteins. The disease progresses over time, presenting with varied and complex pathophysiological changes. In diabetic microangiopathy, endothelial dysfunction plays a central role by impairing the regulation of vascular permeability, cell adhesion, and smooth muscle activity. This dysfunction, marked by reduced vasodilation, chronic inflammation, increased permeability, and leukocyte adhesion, initiates small vessel damage [4]. A key mechanism driving this process is oxidative stress, where the overproduction of reactive oxygen species (ROS) overwhelms the body’s antioxidant defences [5, 6]. This leads to endothelial damage via pathways like the polyol, advanced glycation end products (AGEs), protein kinase C (PKC)-diacylglycerol (DAG), and hexosamine pathways [4]. This disrupts cell junctions and increases vascular permeability, progressively contributing to the breakdown of microvasculature and resulting in complications such as diabetic neuropathy, nephropathy, and retinopathy [4].

Diabetic retinopathy (DR) is a significant complication of diabetes that results from prolonged hyperglycaemia, causing microvascular damage to the retinal vasculature. This leads to leakage of blood and fluid into the retinal tissue, triggering inflammation and the eventual formation of abnormal blood vessels [7]. Over time, these changes culminate in non-proliferative diabetic retinopathy (NPDR) or proliferative diabetic retinopathy (PDR), a more severe form that significantly threatens vision and is a leading cause of blindness worldwide [8]. In India, the overall prevalence of DR is around 16-17%, with urban areas typically showing a slightly higher prevalence (17.4%) compared to rural areas (14.0%) [8]. South India, in particular, has been reported to have a higher prevalence, likely due to a higher burden of diabetes in the region. Age, duration of diabetes, and urbanisation are key factors influencing DR rates [8].

Similarly, diabetic neuropathy (DN) is another microangiopathic manifestation of the disease, causing debilitating bilateral limb pain, numbness, and paraesthesia [9]. The prevalence of DN in India varies significantly across regions due to factors like healthcare access and lifestyle. Urban studies report DN prevalence between 29% and 32.2%, with Bansal et al. noting 29% in Chandigarh [10] and D'Souza et al. reporting 32.2% in Mangalore [11]. Conversely, rural studies reveal much higher rates, such as 52.9% reported by Begum et al. in Puducherry [12] and 51.8% by Vibha et al. in Udupi [13]. The high prevalence and morbidity associated with these chronic diseases necessitate the implementation of efficient and straightforward screening methods to identify patients at an early stage. Early detection is crucial for effectively managing the adverse manifestations of diabetes and improving patient outcomes.

Elevated white blood cell (WBC) count is a recognised inflammatory marker that correlates with various cardiovascular risk factors, diabetes, and its complications [14, 15]. In addition to WBC count, inflammatory cytokines like interleukin (IL)-1, IL-6, IL-8, transforming growth factor β1, and tumour necrosis factor-α are linked to end-organ damage in diabetes [16, 17]. However, these markers face limitations in clinical practice due to availability, cost, and standardisation issues. Among the components of a complete blood count, the neutrophil-lymphocyte ratio (NLR) has gained attention as an inflammatory marker relevant in both cardiac and non-cardiac conditions [18, 19]. NLR serves as a prognostic indicator in acute myocardial infarction, heart failure, and stroke, reflecting a balance between neutrophils, which mediate inflammation, and lymphocytes, which provide regulation and protection. Studies have also shown its significance in early diagnosis of microvascular complications in DM [14, 19, 20]. Despite its potential, data on the role of NLR as a predictor of end-organ damage in Indian patients with T2DM are limited.

The urine albumin-creatinine ratio (UACR) serves as a vital biomarker for assessing kidney function and detecting early signs of diabetic nephropathy. Elevated UACR indicates the presence of albuminuria, often one of the first detectable signs of kidney damage in diabetic patients [21, 22]. American Diabetes Association (ADA) and the National Kidney Foundation (NKF) guidelines define microalbuminuria by an albumin-to-creatinine ratio (ACR) of 3.39-33.9 mg/mmol (30-300 mg/g) [7]. Importantly, increased UACR not only signals potential renal impairment but also serves as a predictive marker for other microvascular complications associated with diabetes. Research has demonstrated a strong association between elevated UACR and the risk of developing DR [7, 23, 24]. Studies indicate that patients with higher levels of albuminuria are more likely to experience progression to both NPDR and PDR. For instance, individuals with microalbuminuria have been shown to have a significantly higher incidence of DR compared to those with normal UACR levels. Furthermore, UACR has also been linked to DN; evidence suggests that patients exhibiting increased albuminuria are at an elevated risk for developing neuropathic symptoms [25, 26].

Given the rising incidence of diabetes and its complications in South Asian populations, particularly in South India, this study aims to investigate whether NLR and UACR are reliable predictors of neuropathy and retinopathy among individuals with type 2 diabetes mellitus in this demographic. By examining these relationships, we seek to contribute valuable insights into early detection strategies for microvascular complications, ultimately enhancing clinical outcomes for patients living with diabetes. The findings from this research could provide essential evidence for the use of NLR and UACR as predictive markers in clinical practice, facilitating timely interventions that may mitigate the impact of these debilitating complications on patients' health and well-being.

## Materials and methods

This cross-sectional study, conducted from August 2022 to February 2024 at Kempegowda Institute of Medical Sciences in Bengaluru, involved patients diagnosed with T2DM who were selected based on predefined inclusion and exclusion criteria to ensure statistical validity. Inclusion criteria comprised individuals aged over 18 years with a confirmed diagnosis of type 2 diabetes mellitus, while patients with type 1 diabetes, recent infections (within the past month), autoimmune disorders, malignancies, haematological disorders, or those taking anti-inflammatory drugs, systemic steroids, or medications affecting the renin-angiotensin-aldosterone system were excluded.

The sample size $N$ was calculated using the formula $N = \frac{Z^2_{(1-\alpha)} \cdot P \cdot Q}{\delta^2}$, where $Z_{(1-\alpha)} = 1.96$ is the Z-value corresponding to a 95% confidence interval, $P = 0.91$ is the estimated sensitivity based on prior research, $Q = 1 - P = 0.09$, and $\delta$ is the margin of error. Using this formula, we calculated a sample size of approximately 130 patients. 

Following the approval from the Institutional Ethics Committee (Ref No.: KIMS/IEC/D054/M/2022, dated 18-07-2022), patients meeting inclusion criteria were enrolled after providing informed consent. A thorough history and physical examination was conducted, recording demographic details (age, sex, height, weight, and duration of diabetes), alongside routine investigations (fasting blood sugar, post-prandial blood sugar, glycosylated haemoglobin (HbA1C), complete blood count and spot UACR), and fundoscopic examination. The NLR, UACR, and other variables were recorded, and their relationship with DN and DR was evaluated. Neuropathy was clinically assessed through patient history and physical examination, while DR was categorised into NPDR and PDR through fundoscopy (grading of NPDR and PDR was not undertaken as a part of this study).

Statistical analysis

Statistical analysis was conducted using SPSS software version 25 (IBM Corp., Armonk, USA). Descriptive statistics, including means and standard deviations for continuous variables and frequencies and percentages for categorical variables, were calculated to summarise the sample characteristics. To assess correlations and differences in the data, ANOVA was employed to compare NLR and UACR across groups defined by the presence or absence of DR, including NPDR and PDR. Independent samples t-tests were used for pairwise comparisons defined by the presence or absence of DN, while chi-square tests examined associations between categorical variables. Receiver-operating characteristic (ROC) curve analysis was also performed to assess the diagnostic performance of NLR and UACR in predicting DN and DR. Additionally, subgroup analyses were conducted to explore variations within specific populations, and logistic regression was used to evaluate the relationship between independent variables and the likelihood of DN and DR.

## Results

Descriptive statistics

The study sample consisted of 130 patients, with a balanced distribution of genders: 55% male (n = 71) and 45% female (n = 59). The mean age of the participants was 57.2 years (SD = 9.09), with a mean HbA1C level of 8.98% (SD = 1.56), indicating poor glycaemic control across the population. The mean duration of diabetes was 6.61 years (SD = 4.84), and the average body mass index (BMI) was 23.24 kg/m² (SD = 2.62).

The NLR had a mean of 2.00 (SD = 0.81), while the UACR averaged 121.00 (SD = 132.72). DN was present in 47.7% of the population (n = 62), and DR was present in 58.5% of the population (n = 76). These results are presented in Tables 1-4.

**Table 1:** Descriptive statistics of patient parameters HbA1c: glycosylated haemoglobin; BMI: body mass index; TLC: total leukocyte count; NLR: neutrophil-lymphocyte Ratio; UACR: urine albumin-creatinine ratio

		Age (years)	Duration of diabetes (years)	HbA1C (%)	TLC (cells/µL)	BMI	NLR	UACR (mg/g)
Mean		57.22	6.61	8.98	7130.62	23.24	1.99	121.00
Median		56.00	5.00	8.80	7135.00	23.00	1.85	60.00
Standard Deviation		9.099	4.84	1.560	1808.18	2.624	.81	132.73
Percentiles	25	50.00	3.00	7.78	5737.50	21.00	1.37	23.75
	50	56.00	5.00	8.80	7135.00	23.00	1.85	60.00
	75	64.00	8.25	10.23	8647.50	25.53	2.39	205.00

**Table 2:** Descriptive statistics of gender distribution

	Number of patients	Percent(%)
Male	71	54.60
Female	59	45.40
Total	130	

**Table 3:** Descriptive statistics of diabetic neuropathy DN: diabetic neuropathy

DN	Number of patients	Percent (%)
Absent	68	52.30
Present	62	47.70
Total	130	

**Table 4:** Descriptive statistics of diabetic retinopathy DR: diabetic retinopathy; NPDR: non-proliferative diabetic retinopathy; PDR: proliferative diabetic retinopathy

DR	Number of patients	Percent (%)
Absent	54	41.50
NPDR	50	38.50
PDR	26	20.00
Total	130	

Independent samples t-test for DN

A paired t-test was conducted to compare the NLR and UACR between patients with and without DN. The results indicated significant differences between the groups. Patients with DN had a significantly higher NLR (M = 3.21) compared to those without DN (M = 2.11), t(128) = 8.40, p < 0.001. Similarly, the UACR was significantly elevated in patients with DN (M = 245.65) compared to those without DN (M = 85.37), t(128) = 9.12, p < 0.001, as seen in Table 5.

**Table 5:** Independent samples t-test comparing means of NLR and UACR in patients with and without DN NLR: neutrophil-lymphocyte ratio; UACR: urine-albumin creatinine ratio; DN: diabetic neuropathy; t: t statistic; dF: degrees of freedom; Sig.: level of significance (p value)

	t	df	Sig.	Mean Difference	Standard Error of Difference	95% confidence interval	
						Lower	Upper
NLR	-8.400	128	.000	-.966	.115	-1.193	-.738
UACR	-9.118	128	.000	-166.108	18.218	-202.155	-130.060

ANOVA for DR with post hoc analysis

A one-way ANOVA with Waller-Duncan post hoc analysis was conducted to compare the means of the NLR and UACR between patients with NPDR and PDR. The results indicated significant differences across the groups, with the mean NLR being significantly higher than normal in the PDR group (mean = 3.86) and the NPDR group (mean = 2.94), F(2, 127) = 69.60, p < 0.001. Similarly, UACR levels increased significantly from the normal group (mean = 25.87) to the NPDR group (mean = 118.78) and the PDR group (mean = 322.85), F(2, 127) = 135.72, p < 0.001, as shown in Tables 6-8.

**Table 6:** ANOVA comparing means of NLR and UACR in patients with and without DR ANOVA: analysis of variance; NLR: neutrophil-lymphocyte ratio; UACR: urine-albumin creatinine ratio; DR: diabetic retinopathy; NPDR: non proliferative diabetic retinopathy; PDR: proliferative diabetic retinopathy; dF: degree of freedom; F: F-statistic; Sig.: level of significance (p value)

		Sum of Squares	df	Mean Square	F	Sig.
NLR	Between Groups	44.492	2	22.246	69.603	.000
	Within Groups	40.591	127	.320		
	Total	85.083	129			
UACR (mg/g)	Between Groups	1548215.943	2	774107.971	135.725	.000
	Within Groups	724344.057	127	5703.497		
	Total	2272560.000	129			

**Table 7:** Waller-Duncan post hoc analysis of NLR in patients with and without DR NLR: neutrophil-lymphocyte ratio; DR: diabetic retinopathy; NPDR: non proliferative diabetic retinopathy; PDR: proliferative diabetic retinopathy

DR	Number of patients	Mean NLR
Absent	54	1.39
NPDR	50	2.17
PDR	26	2.94

**Table 8:** Waller-Duncan post hoc analysis of UACR in patients with and without DR UACR: urine-albumin creatinine ratio; DR: diabetic retinopathy; NPDR: non-proliferative diabetic retinopathy; PDR: proliferative diabetic retinopathy

DR	Number of patients	Mean UACR (mg/g)
Absent	54	25.87
NPDR	50	118.78
PDR	26	322.85

ROC curve for NLR and UACR as predictors of DN and DR

**Figure 1:**
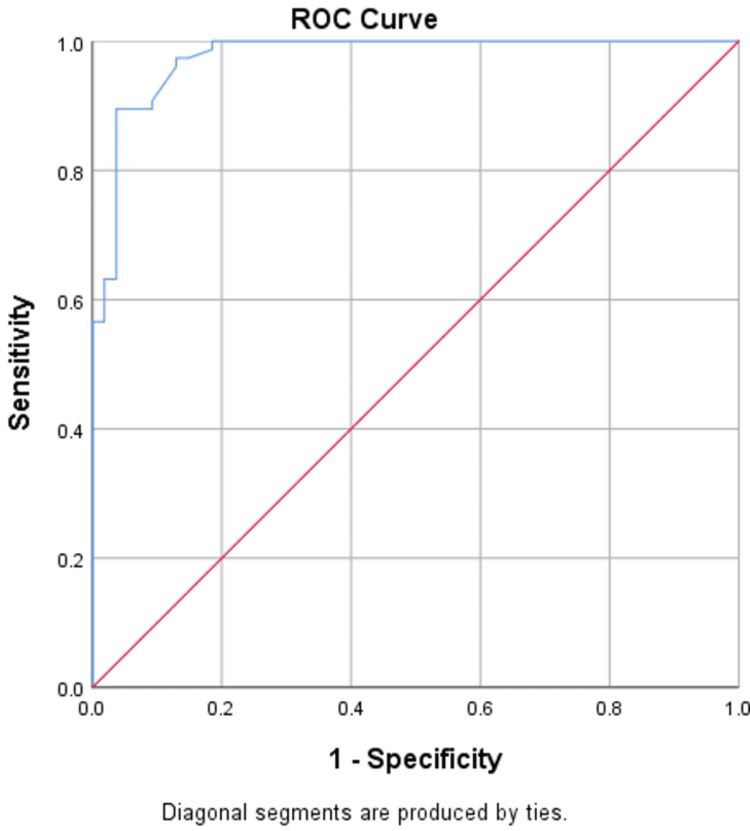
ROC curve of NLR in predicting DN ROC: receiver-operating characteristic; NLR: neutrophil-lymphocyte ratio; DN: diabetic neuropathy

**Figure 2:**
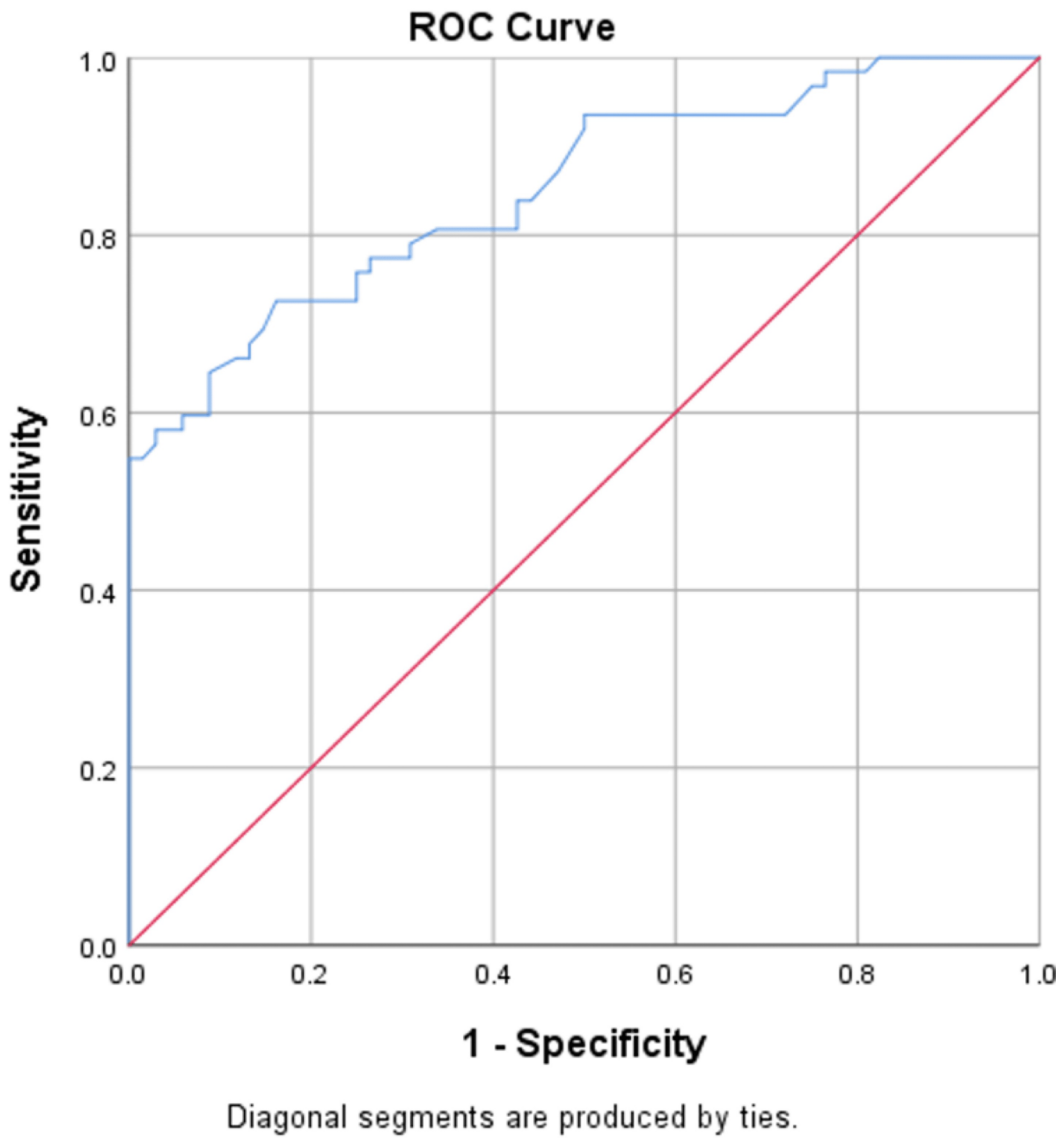
ROC curve of NLR in predicting DR ROC: receiver-operating characteristic; NLR: neutrophil-lymphocyte ratio; DR: diabetic retinopathy

**Figure 3:**
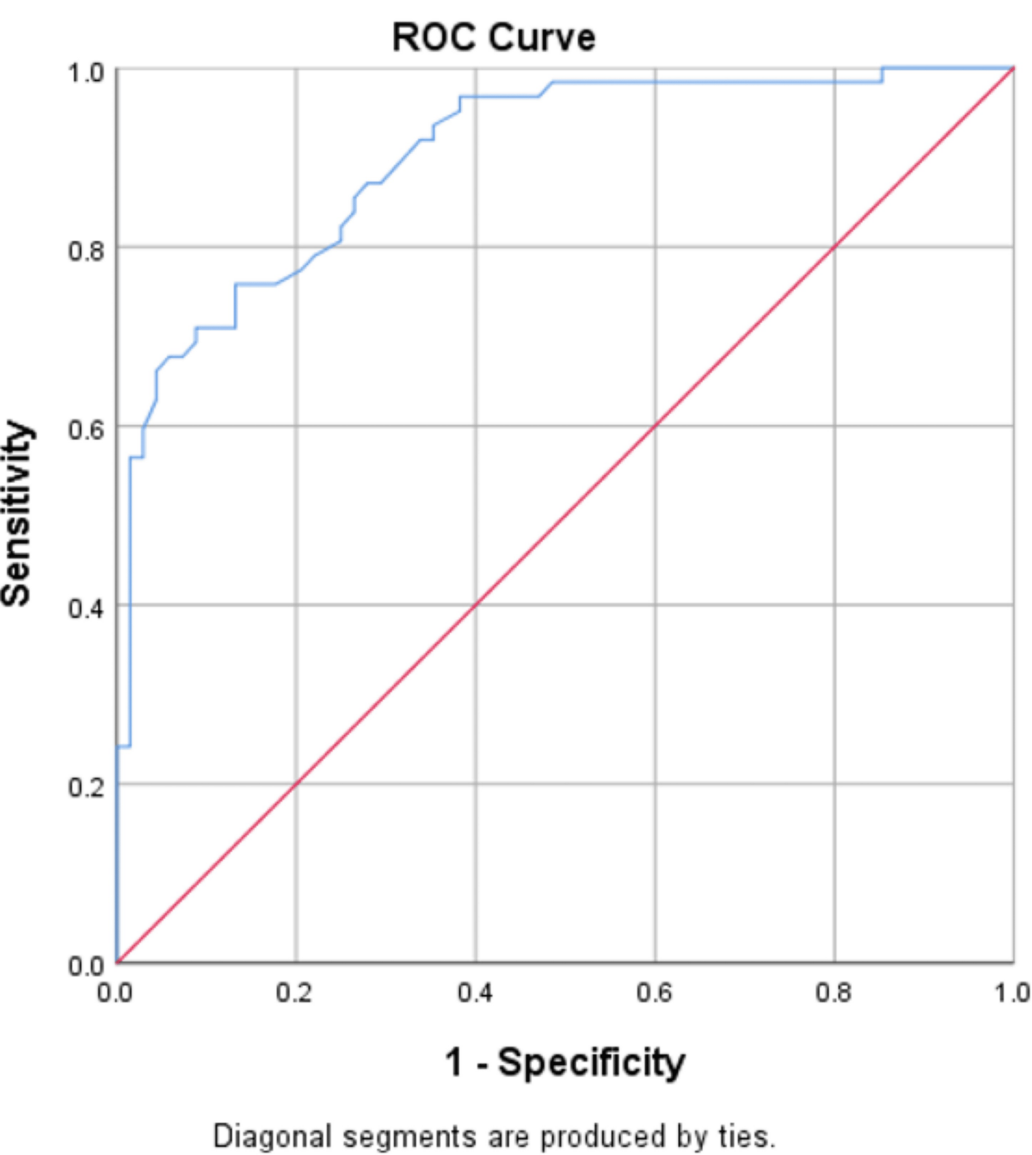
ROC curve of UACR in predicting DN ROC: receiver-operating characteristic; UACR: urine-albumin creatinine ratio; DN: diabetic neuropathy

**Figure 4:**
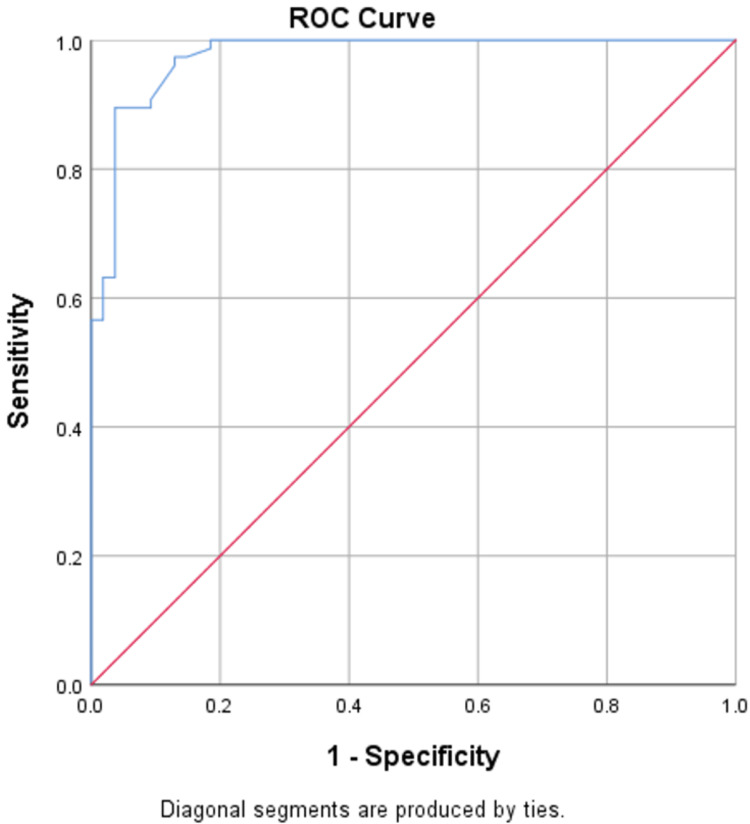
ROC curve of UACR in predicting DR ROC: receiver-operating characteristic; UACR: urine-albumin creatinine ratio; DR: diabetic retinopathy

NLR serves as a reliable independent predictor of both DN and DR, accurately predicting DN in 75% of cases with an area under the curve (AUC) of 0.851, and DR with an accuracy of 85% and an AUC of 0.87, as shown in Figures 1, 2. UACR also serves as a reliable independent predictor of both DN and DR, accurately predicting DN in 81.5% of cases with an AUC of 0.90, and DR with an accuracy of 91% and an AUC of 0.97, as shown in Figures 3, 4.

UACR and NLR quartiles

Patients were divided into quartiles based on the NLR and UACR to examine their associations with diabetic complications. For NLR, the quartiles were as follows: quartile 1 (≤ 1.34), quartile 2 (1.35 - 1.94), quartile 3 (1.95 - 2.43), and quartile 4 (≥ 2.44). For UACR, the quartiles were defined as: quartile 1 (≤ 24 mg/g), quartile 2 (25 - 60 mg/g), quartile 3 (61 - 200 mg/g), and quartile 4 (≥ 201 mg/g).

These quartiles were used to analyse the progression of DN, as well as DR, including NPDR and PDR.

Chi-square analysis of DN and DR across NLR and UACR quartiles

Chi-square tests were conducted to evaluate the relationship between NLR and UACR quartiles and the presence of diabetic nephropathy (DN) and diabetic retinopathy (DR). The results showed strong associations: higher NLR quartiles were significantly linked to DN (χ² = 65.15, p < 0.001), and similarly, higher UACR quartiles were strongly associated with DN (χ² = 75.23, p < 0.001). Additionally, elevated NLR quartiles were significantly related to DR (χ² = 67.99, p < 0.001), as were higher UACR quartiles (χ² = 84.29, p < 0.001). These findings highlight the potential of both biomarkers in identifying patients at risk for diabetic microvascular complications, emphasizing their relevance for early detection and intervention.

**Figure 5:**
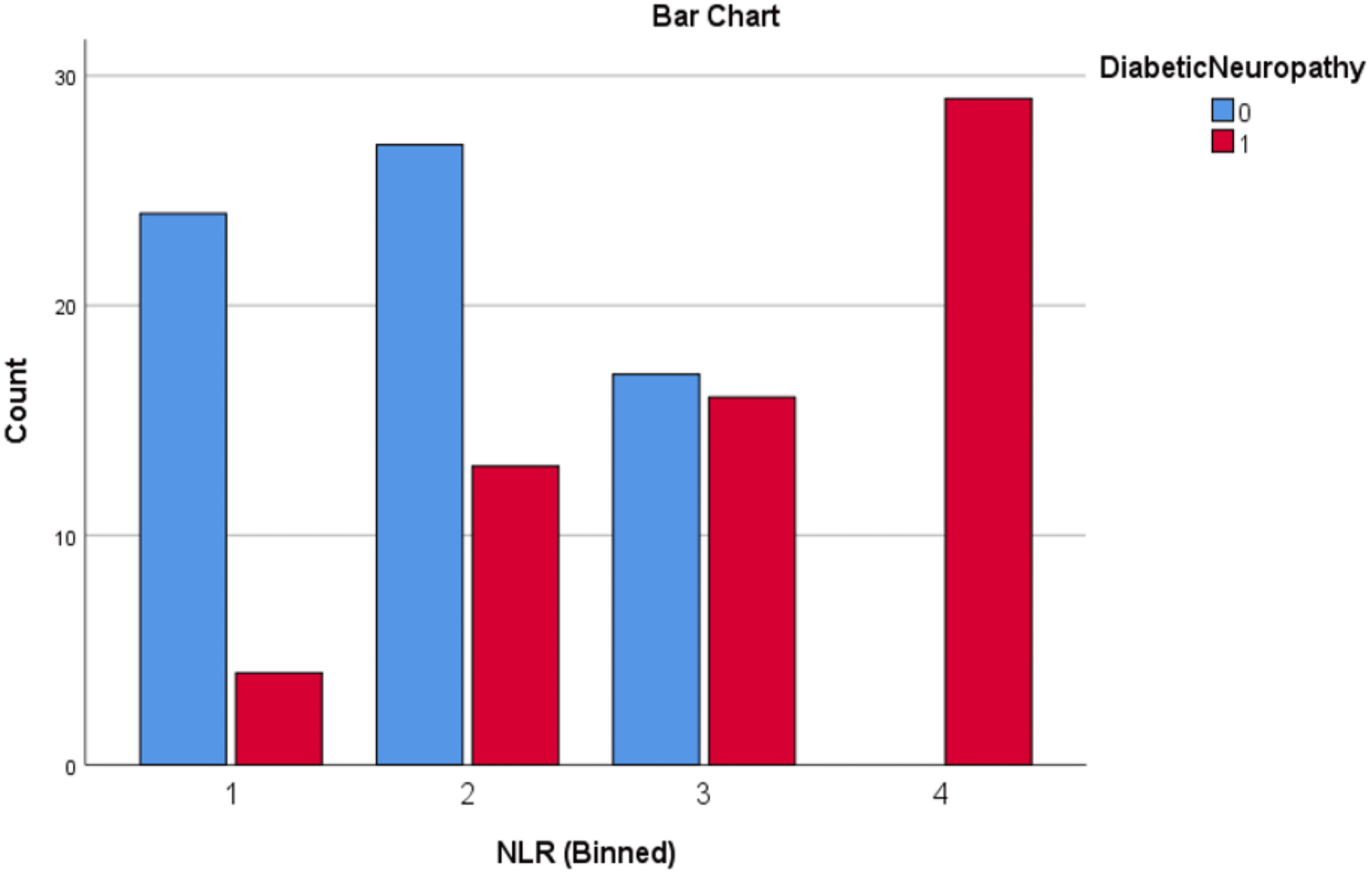
Distribution of DN across NLR quartiles DN: diabetic neuropathy; 0: absent; 1: present; NLR: neutrophil-lymphocyte ratio

**Figure 6:**
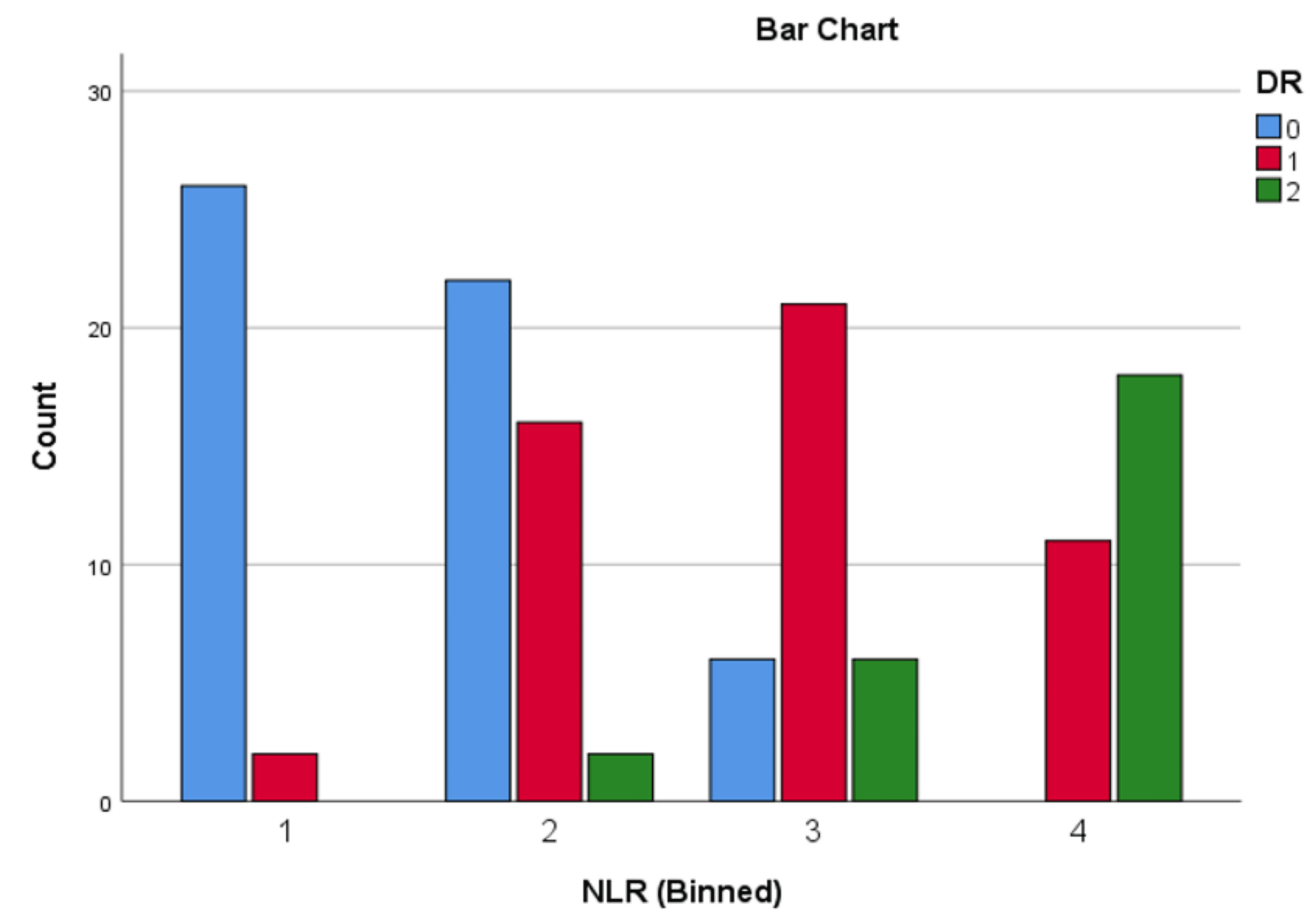
Distribution of DR across NLR quartiles DR: diabetic retinopathy; 0: absent; 1: non-proliferative diabetic retinopathy; 2: proliferative diabetic retinopathy; NLR: neutrophil-lymphocyte ratio

**Figure 7:**
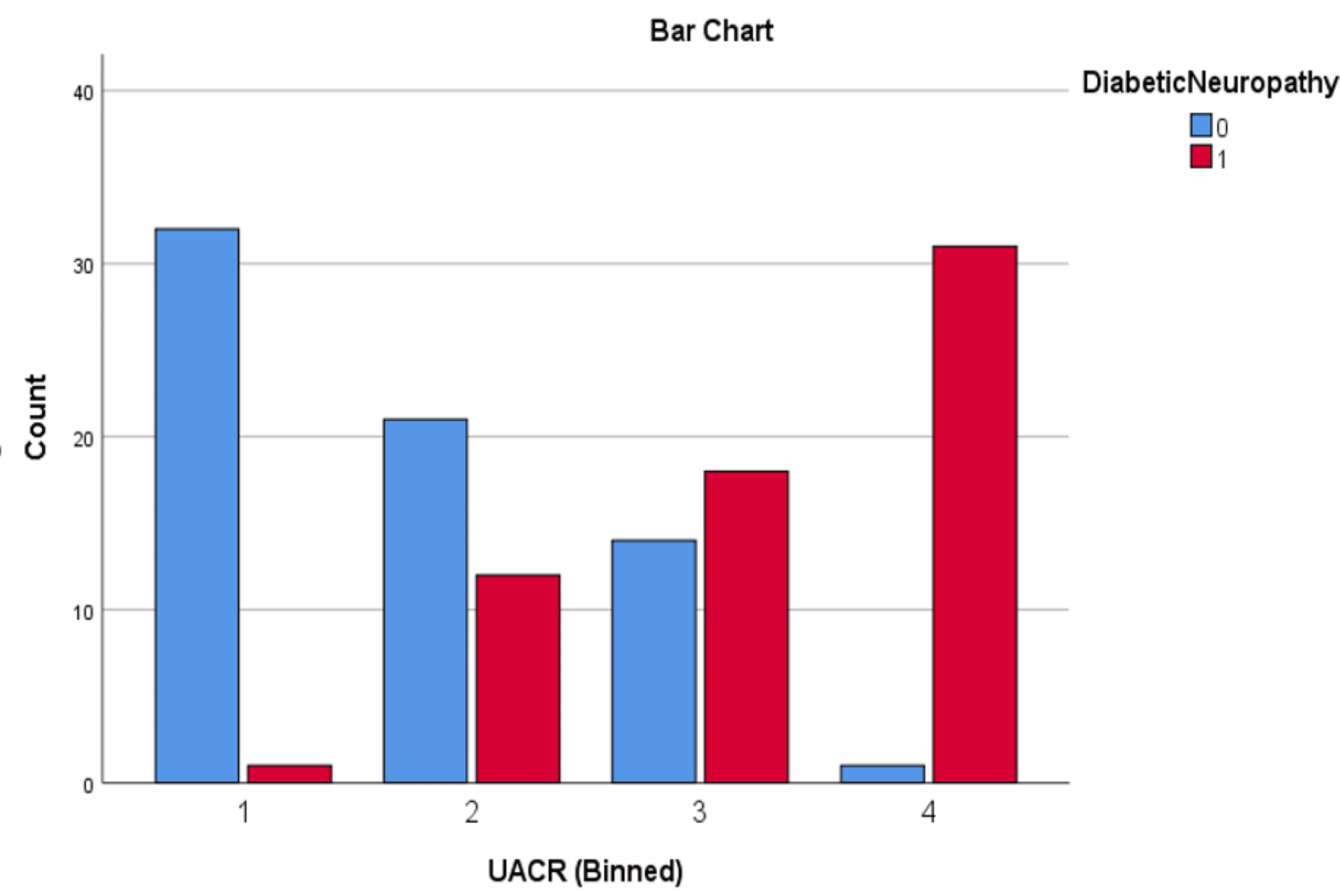
Distribution of DN across UACR quartiles DN: diabetic neuropathy; 0: absent; 1: present; UACR: urine albumin-creatinine ratio

**Figure 8:**
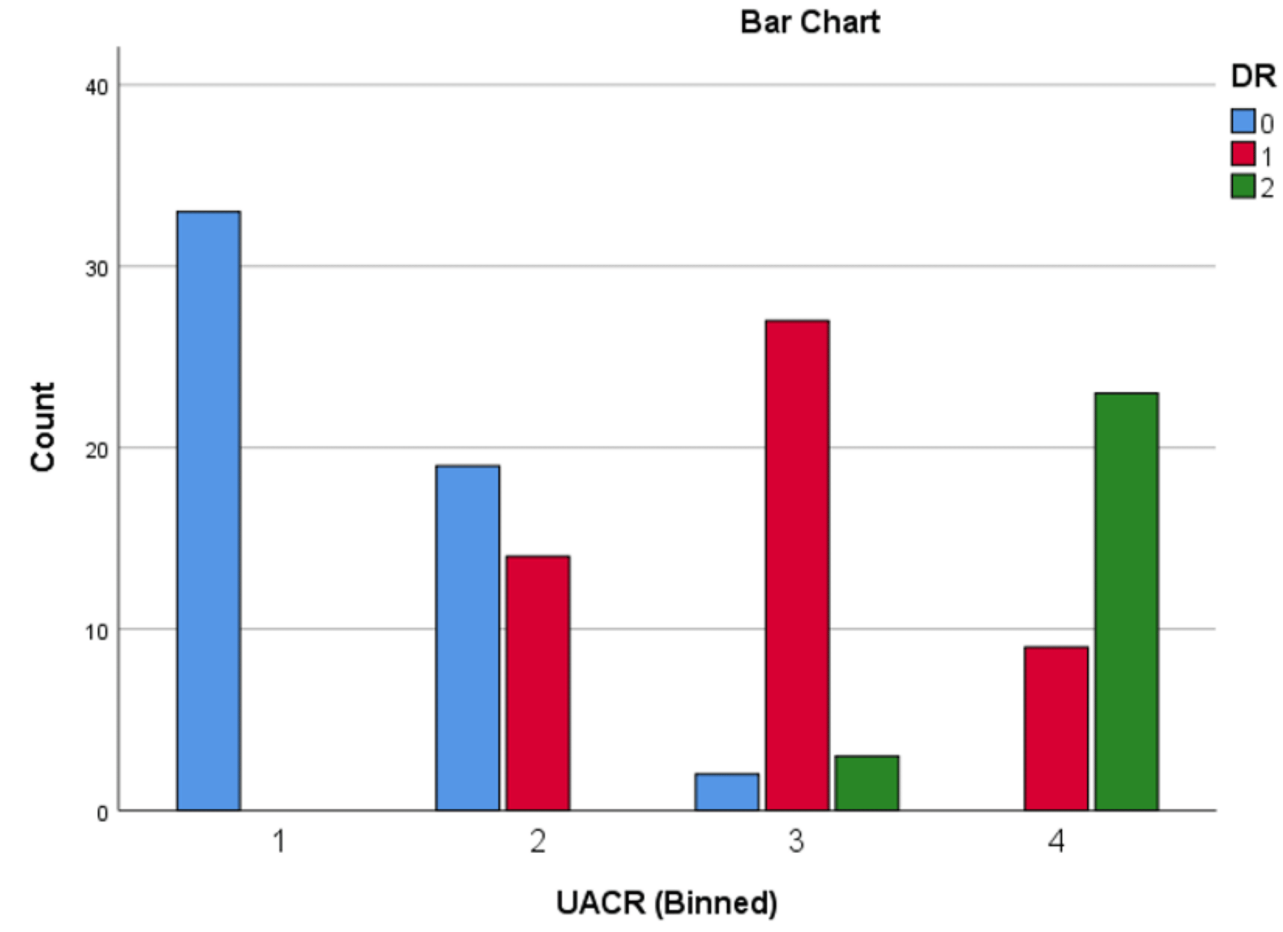
Distribution of DR across UACR quartiles DR: diabetic retinopathy; 0: absent; 1: non-proliferative diabetic retinopathy; 2: proliferative diabetic retinopathy; UACR: urine albumin-creatinine ratio

The distribution of DN and DR across NLR and UACR quartiles can be seen in Figures 5-8.

Logistic regression model for DN

The results of the logistic regression analysis for DN revealed important findings. HbA1C emerged as a strong predictor (B = 0.754, p = 0.002), indicating that higher HbA1C levels more than double the likelihood of DN (OR = 2.126). Similarly, the duration of diabetes was also a significant predictor (B = 0.768, p < 0.001), suggesting that for every unit increase in duration, the odds of developing neuropathy increase by approximately 2.16 times. While other variables, such as age, gender, TLC, BMI, and NLR were included in the model, they were not statistically significant. However, UACR showed a borderline significant effect (p = 0.080). This model provides a robust understanding of the impact of diabetes duration and HbA1C on neuropathy risk, with an overall model fit explaining 75.1% of the variance in the prevalence of DN, as shown in Table 9.

**Table 9:** Logistic regression parameter estimates for diabetic neuropathy DN: diabetic neuropathy; HbA1C: glycosylated haemoglobin; BMI: body mass index; NLR: neutrophil-lymphocyte ratio; UACR: urine albumin-creatinine ratio; TLC: total leukocyte count; B: regression coefficient; dF: degrees of freedom; Sig.: level of significance (p value); Exp(B): exponential regression coefficient/odds ratio; ref.: reference category

Variables	B	Standard Error	Wald Statistic	df	Sig.	Exp(B)
Intercept	-6.836	4.655	2.156	1	.142	
Age	-.099	.061	2.588	1	.108	.906
HbA1C	.754	.242	9.678	1	.002	2.126
Duration Of Diabetes	.768	.207	13.699	1	.000	2.155
BMI	.035	.155	.051	1	.822	1.035
NLR	.668	.778	.738	1	.390	1.951
UACR	.010	.006	3.071	1	.080	1.011
TLC	.000	.000	2.452	1	.117	1.000
Male	.678	.628	1.166	1	.280	1.969
Female	0(ref.)	.	.	0	.	.

Logistic regression model for DR

The multinomial logistic regression model for DR, including NPDR and PDR revealed important findings. The model was able to explain 88.1% of the variance in the prevalence of DR. UACR was a strong predictor of both NPDR and PDR, with a B value of 0.114 (p < 0.001) for NPDR and 0.127 (p < 0.001) for PDR, indicating that each unit increase in UACR raised the odds of NPDR by 12% (OR = 1.120) and PDR by 13.6% (OR = 1.136). The duration of diabetes was a significant predictor for PDR (B = 0.805, OR = 2.238, p = 0.018), suggesting that longer diabetes duration more than doubled the risk of developing PDR. However, it did not significantly predict NPDR. While HbA1C approached significance in the PDR group (B = 0.931, OR = 2.530, p = 0.063), indicating a trend where higher HbA1C values were associated with higher PDR risk, it did not significantly predict NPDR. Other factors, such as age, gender, TLC, BMI, and NLR, were not significant predictors in either category. These findings are shown in Table 10.

**Table 10:** Logistic regression parameter estimates for diabetic retinopathy DR: diabetic retinopathy; NPDR: non-proliferative diabetic retinopathy; PDR: proliferative diabetic retinopathy; HbA1C: glycosylated haemoglobin; BMI: body mass index; NLR: neutrophil-lymphocyte ratio; UACR: urine-albumin creatinine ratio; TLC: total leukocyte count; B: regression coefficient; dF: degrees of freedom; Sig.: level of significance (p value); Exp(B): exponential regression coefficient/odds ratio; ref.: reference category

DR	Variables	B	Standard Error	Wald Statistic	df	Sig.	Exp(B)
NPDR	Intercept	-13.628	8.200	2.762	1	.097	
	Age	.103	.105	.959	1	.327	1.108
	HbA1C	.705	.404	3.050	1	.081	2.52
	Duration Of Diabetes	.530	.304	3.041	1	.081	1.700
	BMI	.134	.274	.241	1	.624	1.144
	NLR	1.481	1.270	1.360	1	.244	4.396
	UACR	.114	.031	13.511	1	.000	1.120
	TLC	.000	.000	.002	1	.963	1.000
	Male	.881	.937	.885	1	.347	2.413
	Female	0(ref.)	.	.	0	.	
PDR	Intercept	-14.744	10.641	1.920	1	.166	
	Age	.078	.128	.376	1	.540	1.081
	HbA1C	.931	.502	3.444	1	.063	2.530
	Duration Of Diabetes	.805	.342	5.549	1	.018	2.238
	BMI	-.003	.359	.000	1	.993	0.997
	NLR	1.569	1.391	1.272	1	.259	4.800
	UACR	.127	.031	16.606	1	.000	1.136
	TLC	.000	.000	.555	1	.456	1.000
	Male	.897	1.227	.534	1	.465	2.452
	Female	0(ref.)	.	.	0	.	

## Discussion

Our study, which investigated the potential of NLR and UACR as predictors of microvascular complications in patients with T2DM, yielded significant findings. The study population (n=130, 54% male, 46% female, mean age of 57.2 years, mean diabetes duration of 6.61 years) exhibited suboptimal glycaemic control (mean HbA1C of 8.98%) and a tendency towards overweight (mean BMI of 23.24 kg/m²). Both NLR and UACR demonstrated strong predictive power for DR and DN. For DN, NLR predicted 75% of cases accurately (AUC of 0.85) and UACR 81.5% (AUC of 0.90). Similarly, for DR, NLR showed 85% accuracy (AUC of 0.87), while UACR demonstrated a 91% accuracy (AUC of 0.97). Statistical analysis using t-tests and ANOVA revealed significant differences in mean NLR and UACR levels between patients with and without DN, as well as across DR subgroups (no DR, NPDR, and PDR) (p < 0.001). Furthermore, patients in the highest quartiles of NLR (≥2.44) and UACR (≥201 mg/g) demonstrated a markedly higher prevalence of both DN and DR compared to lower quartiles, with a particularly strong association with DN and PDR. These results align with previous studies, such as He X et al. [20] and Bhattacharyya S et al. [27], that reported significant correlations between these biomarkers and microvascular complications.

Notably, our study found a high prevalence of nephropathy (66.9% of participants, defined by UACR >30 mg/g). Patients with nephropathy exhibited higher mean NLR (2.30 ± 0.79 vs 1.36 ± 0.33), UACR (171.51 ± 18.81 vs 136.39 ± 6.44 mg/g), and HbA1C (9.45 ± 1.56% vs 8.03 ± 1.05%) compared to those without. These findings align with studies by Kahraman C et al. [28] and Jaaban M et al. [29], further supporting the use of NLR as a surrogate marker for early-stage nephropathy. 

Despite the strong associations seen in ROC-AUC analysis and quartile-based subgroup analysis, logistic regression analysis revealed that NLR was not a statistically significant predictor of DN or DR in multivariate models. For DN, the odds ratio (OR) for NLR was 1.95 (B = 0.668, SE = 0.778, p = 0.390), suggesting a non-significant association. Similarly, for DR, NLR showed high ORs for both NPDR (OR = 4.40, B = 1.481, SE = 1.270, p = 0.244) and PDR (OR = 4.80, B = 1.569, SE = 1.391, p = 0.259), but these associations did not achieve statistical significance. These findings highlight potential issues of limited effect size and the possibility of a type II error due to the relatively small sample size of this study. Furthermore, the predictive power of NLR may be confounded by other unmeasured factors such as acute inflammation, medication use, or comorbidities not systematically accounted for. Alternatively, in the context of a multivariate model incorporating stronger predictors like UACR, duration of diabetes, and HbA1C, NLR may not exert the same level of significance, even though it remains a valuable standalone biomarker.

In contrast, UACR was a significant predictor for DR, particularly for PDR (B = 0.13, OR = 1.13, p < 0.001), and approached significance for NPDR (B = 0.12, OR = 1.12, p = 0.08). However, logistic regression analysis revealed that UACR did not reach statistical significance for DN (B = 0.10, OR = 1.01, p = 0.080), indicating that while UACR may play a role in microvascular health, it may not be a robust predictor for DN specifically.

While gender was not a statistically significant predictor in our study, we observed a slight trend towards higher odds of microvascular complications in males, suggesting the need for further investigation into potential gender-specific risk factors [30]. Importantly, the duration of diabetes emerged as a significant predictor for both DN and PDR, underscoring the critical role of early intervention and sustained glycaemic control in preventing the progression of microvascular complications. Specifically, each additional year of diabetes increased the odds of DN by 115.5% (B = 0.76, OR = 2.15, p < 0.001) and was also a strong predictor of NPDR (B = 0.77, OR = 2.15, p < 0.001). HbA1C similarly showed a strong association with DN, increasing the odds by 112.6% for every percentage point increase (B = 0.75, OR = 2.12, p = 0.002) and approached significance in predicting PDR (B = 0.931, OR = 2.53, p = 0.063).

Despite its strengths, including a comprehensive analysis of multiple microvascular complications, this cross-sectional study has inherent limitations. The single-point data collection restricts our ability to establish causality between NLR, UACR, and the progression of diabetic complications, as cross-sectional designs provide a snapshot in time, identifying associations without establishing temporal relationships. This limitation highlights the need for longitudinal studies that can track patients over time to delineate the dynamic interplay between these biomarkers and disease progression. Although key confounders such as age, HbA1C, BMI, and duration of diabetes were controlled, the potential influence of unmeasured factors, including medication use, comorbidities, and lifestyle habits, cannot be ignored, as these variables may have affected the observed relationships. While efforts were made to control for these factors, the possibility of residual confounding cannot be entirely ruled out due to potential inaccuracies in self-reported data or limitations in capturing all relevant variables. 

Furthermore, the reliance on single measurements of UACR and NLR poses another limitation, as these biomarkers are subject to fluctuations due to temporary changes in kidney function, stress, inflammation, or acute conditions. Repeated measurements over time, as seen in prospective studies, would provide a more reliable assessment of their relationship with diabetic complications. Variations in patient disease severity, healthcare access, and management practices may also influence disease outcomes and the observed associations, highlighting the complexity of these interactions.

Lastly, the geographic and demographic homogeneity of the study population limits the generalisability of the findings, as the cohort consisted predominantly of individuals from a specific region with shared genetic and environmental backgrounds, which may not reflect the diversity of diabetic populations worldwide. Future research should include longitudinal studies to explore causal pathways and better account for temporal variations in biomarker levels, as well as multicentre studies involving diverse populations to validate these findings across different genetic, environmental, and healthcare contexts. Such studies would enhance the applicability of these findings and provide stronger evidence to guide clinical practice and improve patient outcomes.

## Conclusions

In conclusion, our findings, supported by a growing body of literature, strongly reinforce the utility of NLR and UACR as reliable and accessible predictors of diabetic microvascular complications. These easily obtainable and cost-effective biomarkers hold significant potential for improving risk stratification and enabling early, targeted interventions in diabetic care. Their ability to predict severe complications, such as PDR, showcases their clinical value in preventing disease progression and optimizing patient outcomes. Incorporating NLR and UACR into routine clinical practice could greatly enhance the effectiveness of screening protocols, ultimately improving the quality of care and reducing the burden of microvascular complications in diabetes.

However, multinomial logistic regression raises questions about the absolute utility of these variables in predicting microvascular complications, indicating possible confounding factors, multicollinearity among variables, or limited predictive power. Further research is warranted to validate these findings in larger, diverse populations and to explore the potential of these markers in guiding personalized treatment strategies for managing and preventing diabetic microvascular complications.
